# 1-Benzyl-4-(naphthalen-1-yl)-1*H*-1,2,3-triazole

**DOI:** 10.1107/S1600536811019994

**Published:** 2011-06-30

**Authors:** Juan I. Sarmiento-Sánchez, Gerardo Aguirre, Ignacio A. Rivero

**Affiliations:** a Centro de Graduados e Investigación del Instituto Tecnológico de Tijuana, Apdo. Postal 1166, 22500 Tijuana, BC, Mexico

## Abstract

In the title compound, C_19_H_15_N_3_, the benzyl group is almost perpendicular to the triazole ring [dihedral angle = 80.64 (8)°], while the napthyl group makes an angle of 30.27 (12)° with the plane of the triazole ring. This conformation is different from the 1-benzyl-4-phenyl-1*H*-1,2,3-triazole analogue, which has the benzyl ring system at an angle of 87.94° and the phenyl group at an angle of 3.35° to the plane of the triazole ring.

## Related literature

For the biological activity of triazoles, see: Alvarez *et al.* (1994 ▶ **);** Brockunier *et al.* (2000 ▶); Genin *et al.* (2000 ▶); Katritsky *et al.* (1996 ▶ **).** For related structures, see: Bi (2010 ▶); Huang *et al.* (2010 ▶); Jabli *et al.* (2010 ▶); Key *et al.* (2008 ▶); Makam & Yulin (2004 ▶); Santos-Contreras *et al.* (2009 ▶): Vaqueiro (2006 ▶).
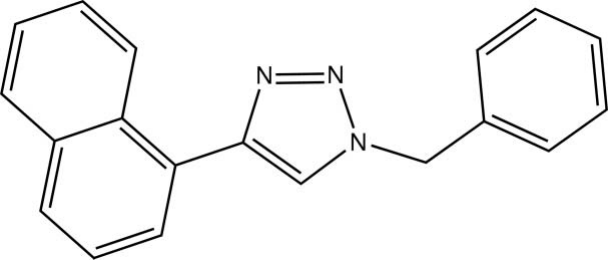


## Experimental

### 

#### Crystal data

C_19_H_15_N_3_
*M* *_r_* = 285.34
Monoclinic, 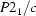

*a* = 9.896 (2) Å
*b* = 11.038 (3) Å
*c* = 14.136 (4) Å
β = 102.701 (13)°
*V* = 1506.2 (6) Å^3^
*Z* = 4
Mo *K*α radiation
μ = 0.08 mm^−1^
*T* = 298 K
0.5 × 0.48 × 0.28 mm

#### Data collection

Siemens P4 diffractometer
3663 measured reflections
3471 independent reflections
1730 reflections with *I* > 2σ(*I*)
*R*_int_ = 0.028
3 standard reflections every 97 reflections  intensity decay: 5.4%

#### Refinement

*R*[*F* ^2^ > 2σ(*F* ^2^)] = 0.069
*wR*(*F* ^2^) = 0.202
*S* = 1.01
3471 reflections
199 parameters
H-atom parameters constrained
Δρ_max_ = 0.40 e Å^−3^
Δρ_min_ = −0.17 e Å^−3^

### 

Data collection: *XSCANS* (Siemens, 1996 ▶); cell refinement: *XSCANS*; data reduction: *XSCANS*; program(s) used to solve structure: *SHELXS97* (Sheldrick, 2008 ▶); program(s) used to refine structure: *SHELXL97* (Sheldrick, 2008 ▶); molecular graphics: *Mercury* (Macrae *et al.*, 2006 ▶); software used to prepare material for publication: *SHELXL97*.

## Supplementary Material

Crystal structure: contains datablock(s) I, global. DOI: 10.1107/S1600536811019994/fl2334sup1.cif

Structure factors: contains datablock(s) I. DOI: 10.1107/S1600536811019994/fl2334Isup2.hkl

Supplementary material file. DOI: 10.1107/S1600536811019994/fl2334Isup3.cml

Additional supplementary materials:  crystallographic information; 3D view; checkCIF report

## Figures and Tables

**Fig. 1.**
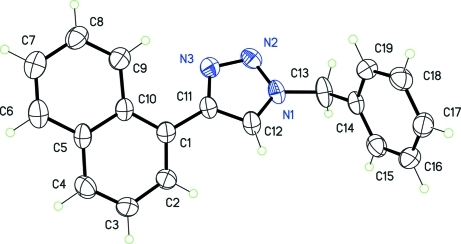
The title compound (I) with displacement ellipsoids drawn at 30% probability level.

## supplementary crystallographic information

### Comment

In recent years, triazole compounds have received much attention due to their wide range of applications in organic and medicinal chemistry. Specifically, 1,2,3-triazoles have been used in pharmaceuticals, agrochemicals, dyes, photographic materials and corrosion inhibitors (Katritsky *et al.*, 1996). There are numerous examples in the literature of the biological activity of triazole compounds acting as as anti-HIV agents (Alvarez *et al.*, 1994) or as antibiotics due to their antimicrobial activity against Gram positive bacteria (Genin *et al.*, 2000) and as selective β_3_ adrenergic agonist receptors (Brockunier *et al.*, 2000).

The molecular structure of (I) is shown in Fig. 1. The molecule shows that the phenyl group and the triazole heterocycle are linked by the methylene group. The carbon atom C13 with a C14—C13—N1 angle of 112.5 (2)*^o^* is distorted from ideal tetrahedral geometry (109.7¯). This can be attributed to steric factors of adjacent cyclic systems. Also, the bonds distances N3—C11, C11—C12, C12—N1, N1—N2 and N2—N3 are 1.353 (3), 1.353 (4), 1.335 (3), 1.337 (3) and 1.321 (3) Å, respectively, which agree with the C═C, N═N and C—N distances found in the literature for compounds having triazole heterocycles (Huang *et al.*, 2010; Jabli *et al.*, 2010; Key *et al.* 2008). In addition, C12—N1 and C11—N3 are significantly shorter that the C—N single bonds (1.47 Å) (Vaqueiro, 2006; Bi, 2010) but longer than true C—N double bonds (1.28 Å) (Santos-Contreras *et al.*, 2009). This indicates a delocalization of electrons in the triazolyl system.

As shown in Fig. 1, the molecule shows the benzyl group is located above the plane of the triazole at a dihedral angle of 80.64 (0.08)° and the naphthyl group is at an angle fo 30.27 (0.12)°. This conformation is different from its analogue 1-benzyl-4-phenyl-1*H*-1, 2,3-triazole which presents the benzyl at an dihedral angle of 87.94° and the phenyl at an angle of 3.35° to the plane of the triazole (Makam & Yulin, 2004).

### Experimental

Experimental

All reagents were purchased in the highest quality available and were used without further purification. The solvents used in column chromatography were obtained from commercial suppliers and used without distillation. To a solution *tert*-BuOH/H_2_O (6 ml 1:1 *v*/*v*) was added benzyl bromide (1.684 mmol), sodium azide (1.684 mmol), 1-ethynyl-naphthalene (1.684 mmol), copper(II) sulfate (0.084 mmol, 5% mol) and sodium ascorbate (0.168 mmol, 10% mol) with vigorous stirring at 60 °C for 8 h. The reaction mixture was filtered with diatomaceous earth (kieselguhr) or zeolite and silica gel in vacuo, then extracted with ethyl acetate (60 ml). The extracts were combined and dried over anhydrous sodium sulfate. After evaporation of the solvent, the residual oil solidified and was purified by flash chromatography to give (I) (petroleum ether/EtOAc 1:1 *v*/*v*). Yield 85%; pale yellow solid; mp 89–90 °C; ^1^H-NMR (CDCl_3_, 200 MHz): δ 8.39–8.34 (m, 1H), 7.88–7.82 (m, 2H), 7.71 (s, 1H), 7.69–7.66 (d, *J* = 7.33 Hz, 1H), 7.52–7.47 (dd, *J* = 6.42, 3.48 Hz, 4H), 7.37–7.36 (d, *J* = 1.83 Hz, 4H), 5.61 (s, 2H); ^13^C-NMR (CDCl_3_, 50 MHz): δ 147.3, 134.6, 133.8, 131.0, 129.1, 128.8, 128.7, 128.3, 128.0, 127.1, 126.5, 125.9, 125.4, 125.2, 122.4, 54.1; IR (KBr, pellet): 1686, 1601, 1454 cm^-1^; ESI-MS *m/z*: 286 [*M*+H]^+^, 308 [*M*+Na]^+^, 324 [*M*+K]^+^, 593 [2*M*+Na]^+^.

Crystallization

50 mg of (I) compound was placed for diffusion in a glass vial with chloroform-petroleum ether for one day. The crystals, suitable for data collection, were separated by filtration.

### Refinement

Refinement for H atoms was carried out using a riding model, with distances constrained to: 0.93 Å for aromatic CH, 0.98 Å for methine CH. Isotropic U parameters were fixed to *U*_iso_(H)=1.2*U*_eq_(carrier atom) for aromatic CH.

### Crystal data

**Table:**

C_19_H_15_N_3_	*F*(000) = 600
*M**_r_* = 285.34	*D*_x_ = 1.258 Mg m^−^^3^
Monoclinic, *P*2_1_/*c*	Mo *K*α radiation, λ = 0.71073 Å
Hall symbol: -P 2ybc	Cell parameters from 36 reflections
*a* = 9.896 (2) Å	θ = 4.6–12.4°
*b* = 11.038 (3) Å	µ = 0.08 mm^−^^1^
*c* = 14.136 (4) Å	*T* = 298 K
β = 102.701 (13)°	Prismatic, colorless
*V* = 1506.2 (6) Å^3^	0.5 × 0.48 × 0.28 mm
*Z* = 4	

### Data collection

**Table:**

Siemens P4 diffractometer	*R*_int_ = 0.028
Radiation source: fine-focus sealed tube	θ_max_ = 27.5°, θ_min_ = 2.1°
graphite	*h* = 0→12
2θ/ω scans	*k* = 0→14
3663 measured reflections	*l* = −18→17
3471 independent reflections	3 standard reflections every 97 reflections
1730 reflections with *I* > 2σ(*I*)	intensity decay: 5.4%

### Refinement

**Table:**

Refinement on *F*^2^	Primary atom site location: structure-invariant direct methods
Least-squares matrix: full	Secondary atom site location: difference Fourier map
*R*[*F*^2^ > 2σ(*F*^2^)] = 0.069	Hydrogen site location: inferred from neighbouring sites
*wR*(*F*^2^) = 0.202	H-atom parameters constrained
*S* = 1.01	*w* = 1/[σ^2^(*F*_o_^2^) + (0.0839*P*)^2^ + 0.308*P*] where *P* = (*F*_o_^2^ + 2*F*_c_^2^)/3
3471 reflections	(Δ/σ)_max_ < 0.001
199 parameters	Δρ_max_ = 0.40 e Å^−^^3^
0 restraints	Δρ_min_ = −0.17 e Å^−^^3^

### Special details

**Table:**

Geometry. All e.s.d.'s (except the e.s.d. in the dihedral angle between two l.s. planes) are estimated using the full covariance matrix. The cell e.s.d.'s are taken into account individually in the estimation of e.s.d.'s in distances, angles and torsion angles; correlations between e.s.d.'s in cell parameters are only used when they are defined by crystal symmetry. An approximate (isotropic) treatment of cell e.s.d.'s is used for estimating e.s.d.'s involving l.s. planes.
Refinement. Refinement of *F*^2^ against ALL reflections. The weighted *R*-factor *wR* and goodness of fit *S* are based on *F*^2^, conventional *R*-factors *R* are based on *F*, with *F* set to zero for negative *F*^2^. The threshold expression of *F*^2^ > σ(*F*^2^) is used only for calculating *R*-factors(gt) *etc*. and is not relevant to the choice of reflections for refinement. *R*-factors based on *F*^2^ are statistically about twice as large as those based on *F*, and *R*- factors based on ALL data will be even larger.

### Fractional atomic coordinates and isotropic or equivalent isotropic displacement parameters (Å^2^)

**Table:**

	*x*	*y*	*z*	*U*_iso_*/*U*_eq_	
N1	0.2899 (2)	0.3968 (2)	0.18018 (16)	0.0603 (7)	
N2	0.3058 (3)	0.2778 (3)	0.19682 (18)	0.0688 (7)	
N3	0.4044 (3)	0.2660 (2)	0.27619 (18)	0.0665 (7)	
C1	0.5662 (3)	0.4031 (3)	0.39312 (19)	0.0548 (7)	
C2	0.6434 (3)	0.5043 (3)	0.3915 (2)	0.0688 (9)	
H2B	0.6208	0.5550	0.3377	0.083*	
C3	0.7552 (4)	0.5365 (3)	0.4664 (2)	0.0779 (10)	
H3B	0.8053	0.6066	0.4613	0.093*	
C4	0.7905 (4)	0.4661 (3)	0.5459 (2)	0.0773 (10)	
H4A	0.8655	0.4870	0.5954	0.093*	
C5	0.7114 (3)	0.3581 (3)	0.55387 (19)	0.0592 (8)	
C6	0.7469 (4)	0.2850 (3)	0.6363 (2)	0.0756 (10)	
H6A	0.8207	0.3068	0.6864	0.091*	
C7	0.6745 (4)	0.1828 (3)	0.6434 (2)	0.0779 (10)	
H7A	0.6995	0.1339	0.6981	0.093*	
C8	0.5616 (3)	0.1501 (3)	0.5686 (2)	0.0726 (9)	
H8A	0.5123	0.0799	0.5748	0.087*	
C9	0.5231 (3)	0.2188 (3)	0.4877 (2)	0.0657 (8)	
H9A	0.4475	0.1958	0.4394	0.079*	
C10	0.5991 (3)	0.3282 (2)	0.47587 (18)	0.0519 (7)	
C11	0.4501 (3)	0.3772 (3)	0.30902 (19)	0.0530 (7)	
C12	0.3770 (3)	0.4599 (3)	0.2479 (2)	0.0627 (8)	
H12A	0.3856	0.5438	0.2521	0.075*	
C13	0.1908 (3)	0.4396 (4)	0.0935 (2)	0.0837 (11)	
H13A	0.1334	0.3723	0.0644	0.100*	
H13B	0.2413	0.4688	0.0465	0.100*	
C14	0.0994 (3)	0.5392 (3)	0.11590 (18)	0.0559 (7)	
C15	0.1201 (3)	0.6574 (3)	0.0925 (2)	0.0713 (9)	
H15A	0.1931	0.6762	0.0634	0.086*	
C16	0.0351 (4)	0.7485 (3)	0.1112 (2)	0.0768 (10)	
H16A	0.0509	0.8280	0.0946	0.092*	
C17	−0.0723 (3)	0.7230 (3)	0.1539 (2)	0.0687 (9)	
H17A	−0.1301	0.7846	0.1664	0.082*	
C18	−0.0945 (3)	0.6056 (3)	0.1783 (2)	0.0680 (9)	
H18A	−0.1673	0.5875	0.2078	0.082*	
C19	−0.0089 (3)	0.5140 (3)	0.1592 (2)	0.0617 (8)	
H19A	−0.0247	0.4345	0.1759	0.074*	

### Atomic displacement parameters (Å^2^)

**Table:**

	*U*^11^	*U*^22^	*U*^33^	*U*^12^	*U*^13^	*U*^23^
N1	0.0498 (14)	0.0771 (18)	0.0486 (13)	0.0138 (13)	−0.0007 (11)	−0.0144 (12)
N2	0.0644 (16)	0.0719 (19)	0.0660 (16)	−0.0103 (14)	0.0052 (13)	−0.0132 (14)
N3	0.0671 (16)	0.0661 (17)	0.0624 (15)	−0.0031 (13)	0.0060 (13)	0.0003 (13)
C1	0.0597 (17)	0.0507 (17)	0.0522 (16)	0.0095 (14)	0.0082 (13)	−0.0086 (13)
C2	0.078 (2)	0.0553 (19)	0.0652 (19)	−0.0055 (17)	−0.0020 (16)	−0.0066 (15)
C3	0.093 (2)	0.0549 (19)	0.075 (2)	−0.0171 (17)	−0.0047 (19)	−0.0025 (17)
C4	0.083 (2)	0.069 (2)	0.068 (2)	−0.0057 (18)	−0.0085 (18)	−0.0155 (18)
C5	0.0637 (18)	0.0638 (19)	0.0460 (15)	0.0207 (15)	0.0033 (14)	−0.0046 (13)
C6	0.078 (2)	0.086 (3)	0.0567 (18)	0.023 (2)	0.0022 (17)	−0.0063 (17)
C7	0.084 (2)	0.083 (3)	0.067 (2)	0.021 (2)	0.0194 (19)	0.0096 (18)
C8	0.076 (2)	0.076 (2)	0.071 (2)	0.0046 (18)	0.0274 (18)	0.0163 (17)
C9	0.0626 (19)	0.069 (2)	0.0681 (19)	0.0069 (16)	0.0205 (15)	0.0056 (16)
C10	0.0551 (16)	0.0530 (16)	0.0463 (14)	0.0164 (14)	0.0085 (13)	−0.0066 (12)
C11	0.0507 (15)	0.0598 (18)	0.0459 (14)	0.0107 (13)	0.0049 (12)	−0.0105 (13)
C12	0.0637 (18)	0.0594 (18)	0.0568 (16)	0.0166 (15)	−0.0043 (14)	−0.0165 (14)
C13	0.068 (2)	0.126 (3)	0.0482 (17)	0.034 (2)	−0.0059 (15)	−0.0121 (18)
C14	0.0457 (15)	0.076 (2)	0.0414 (14)	0.0091 (14)	−0.0001 (12)	0.0008 (14)
C15	0.0512 (18)	0.098 (3)	0.0633 (19)	−0.0065 (18)	0.0092 (15)	0.0198 (18)
C16	0.080 (2)	0.067 (2)	0.074 (2)	−0.0104 (19)	−0.0025 (19)	0.0197 (17)
C17	0.068 (2)	0.063 (2)	0.0697 (19)	0.0095 (16)	0.0046 (17)	0.0042 (16)
C18	0.0528 (18)	0.081 (2)	0.075 (2)	−0.0010 (17)	0.0227 (16)	0.0033 (17)
C19	0.0641 (18)	0.0548 (18)	0.0645 (18)	−0.0021 (15)	0.0109 (15)	0.0042 (14)

### Geometric parameters (Å, °)

**Table:**

N1—C12	1.335 (3)	C8—C9	1.356 (4)
N1—N2	1.337 (3)	C8—H8A	0.9300
N1—C13	1.470 (3)	C9—C10	1.452 (4)
N2—N3	1.321 (3)	C9—H9A	0.9300
N3—C11	1.353 (3)	C11—C12	1.353 (4)
C1—C2	1.357 (4)	C12—H12A	0.9300
C1—C10	1.410 (4)	C13—C14	1.500 (4)
C1—C11	1.488 (4)	C13—H13A	0.9700
C2—C3	1.399 (4)	C13—H13B	0.9700
C2—H2B	0.9300	C14—C15	1.372 (4)
C3—C4	1.348 (4)	C14—C19	1.375 (4)
C3—H3B	0.9300	C15—C16	1.374 (5)
C4—C5	1.444 (5)	C15—H15A	0.9300
C4—H4A	0.9300	C16—C17	1.363 (5)
C5—C6	1.397 (4)	C16—H16A	0.9300
C5—C10	1.421 (4)	C17—C18	1.371 (4)
C6—C7	1.352 (5)	C17—H17A	0.9300
C6—H6A	0.9300	C18—C19	1.384 (4)
C7—C8	1.407 (5)	C18—H18A	0.9300
C7—H7A	0.9300	C19—H19A	0.9300
			
C12—N1—N2	110.8 (2)	C1—C10—C9	123.5 (3)
C12—N1—C13	129.6 (3)	C5—C10—C9	116.1 (3)
N2—N1—C13	119.6 (3)	C12—C11—N3	107.6 (2)
N3—N2—N1	106.4 (2)	C12—C11—C1	126.2 (3)
N2—N3—C11	109.3 (2)	N3—C11—C1	126.1 (3)
C2—C1—C10	117.9 (3)	N1—C12—C11	106.0 (3)
C2—C1—C11	118.9 (3)	N1—C12—H12A	127.0
C10—C1—C11	123.2 (3)	C11—C12—H12A	127.0
C1—C2—C3	123.4 (3)	N1—C13—C14	112.5 (2)
C1—C2—H2B	118.3	N1—C13—H13A	109.1
C3—C2—H2B	118.3	C14—C13—H13A	109.1
C4—C3—C2	120.1 (3)	N1—C13—H13B	109.1
C4—C3—H3B	120.0	C14—C13—H13B	109.1
C2—C3—H3B	120.0	H13A—C13—H13B	107.8
C3—C4—C5	119.7 (3)	C15—C14—C19	118.1 (3)
C3—C4—H4A	120.1	C15—C14—C13	121.1 (3)
C5—C4—H4A	120.1	C19—C14—C13	120.7 (3)
C6—C5—C10	121.6 (3)	C14—C15—C16	121.4 (3)
C6—C5—C4	120.0 (3)	C14—C15—H15A	119.3
C10—C5—C4	118.4 (3)	C16—C15—H15A	119.3
C7—C6—C5	120.3 (3)	C17—C16—C15	120.2 (3)
C7—C6—H6A	119.9	C17—C16—H16A	119.9
C5—C6—H6A	119.9	C15—C16—H16A	119.9
C6—C7—C8	120.3 (3)	C16—C17—C18	119.4 (3)
C6—C7—H7A	119.9	C16—C17—H17A	120.3
C8—C7—H7A	119.9	C18—C17—H17A	120.3
C9—C8—C7	121.4 (3)	C17—C18—C19	120.2 (3)
C9—C8—H8A	119.3	C17—C18—H18A	119.9
C7—C8—H8A	119.3	C19—C18—H18A	119.9
C8—C9—C10	120.4 (3)	C14—C19—C18	120.7 (3)
C8—C9—H9A	119.8	C14—C19—H19A	119.7
C10—C9—H9A	119.8	C18—C19—H19A	119.7
C1—C10—C5	120.4 (3)		
			
C12—N1—N2—N3	0.0 (3)	C8—C9—C10—C5	−1.2 (4)
C13—N1—N2—N3	177.3 (2)	N2—N3—C11—C12	0.1 (3)
N1—N2—N3—C11	−0.1 (3)	N2—N3—C11—C1	−176.1 (2)
C10—C1—C2—C3	1.4 (5)	C2—C1—C11—C12	−27.0 (4)
C11—C1—C2—C3	−179.5 (3)	C10—C1—C11—C12	151.9 (3)
C1—C2—C3—C4	−0.6 (5)	C2—C1—C11—N3	148.4 (3)
C2—C3—C4—C5	−0.7 (5)	C10—C1—C11—N3	−32.6 (4)
C3—C4—C5—C6	−179.7 (3)	N2—N1—C12—C11	0.1 (3)
C3—C4—C5—C10	1.1 (5)	C13—N1—C12—C11	−176.9 (3)
C10—C5—C6—C7	0.2 (5)	N3—C11—C12—N1	−0.1 (3)
C4—C5—C6—C7	−179.0 (3)	C1—C11—C12—N1	176.0 (2)
C5—C6—C7—C8	−0.9 (5)	C12—N1—C13—C14	−50.4 (4)
C6—C7—C8—C9	0.5 (5)	N2—N1—C13—C14	132.8 (3)
C7—C8—C9—C10	0.6 (5)	N1—C13—C14—C15	104.8 (3)
C2—C1—C10—C5	−1.0 (4)	N1—C13—C14—C19	−76.2 (4)
C11—C1—C10—C5	−179.9 (2)	C19—C14—C15—C16	−0.3 (4)
C2—C1—C10—C9	178.7 (3)	C13—C14—C15—C16	178.7 (3)
C11—C1—C10—C9	−0.2 (4)	C14—C15—C16—C17	0.1 (5)
C6—C5—C10—C1	−179.5 (3)	C15—C16—C17—C18	0.2 (5)
C4—C5—C10—C1	−0.3 (4)	C16—C17—C18—C19	−0.3 (5)
C6—C5—C10—C9	0.8 (4)	C15—C14—C19—C18	0.2 (4)
C4—C5—C10—C9	−180.0 (3)	C13—C14—C19—C18	−178.8 (3)
C8—C9—C10—C1	179.1 (3)	C17—C18—C19—C14	0.1 (5)

## References

[bb1] Alvarez, R., Elazquez, S. V., San, F., Aquaro, S., De, C., Perno, C. F., Karlesson, A., Balzarini, J. & Camarasa, M. J. (1994). *J. Med. Chem.* **37**, 4185–4194. 10.1021/jm00050a015 7527463

[bb2] Bi, Y. (2010). *Acta Cryst.* E**66**, o951. 10.1107/S1600536810010846 PMC2984010 21580755

[bb3] Brockunier, L. L., Parmee, E. R., Ok, H. O., Candelore, M. R., Cascieri, M. A., Colwell, L. F., Deng, L., Feeney, W. P., Forest, M. J., Hom, G. J., MacIntyre, D. E., Tota, L., Wyvratt, M. J., Fisher, M. H. & Weber, A. E. (2000). *Bioorg. Med. Chem. Lett.* **10**, 2111–2114. 10.1016/s0960-894x(00)00422-4 10999482

[bb4] Genin, M. J., Allwine, D. A., Anderson, D. J., Barbachyn, M. R., Emmert, D. E., Garmon, S. A., Graber, D. R., Grega, K. C., Hester, J. B., Hutchinson, D. K., Morris, J., Reischer, R. J., Ford, C. W., Zurenco, G. E., Hamel, J. C., Schaadt, R. D., Stapertand, D. & Yagi, B. H. (2000). *J. Med. Chem.* **43**, 953–970. 10.1021/jm990373e 10715160

[bb5] Huang, C.-C., Wu, F.-L., Lo, Y. H., Lai, W.-R. & Lin, C.-H. (2010). *Acta Cryst.* E**66**, o1690. 10.1107/S1600536810022531 PMC3006897 21587913

[bb6] Jabli, H., Ouazzani Chahdi, F., Saffon, N., Essassi, E. M. & Ng, S. W. (2010). *Acta Cryst.* E**66**, o232. 10.1107/S1600536809054464 PMC2980277 21580114

[bb7] Katritsky, A. R., Rees, C. W. & Scriven, C. W. V. (1996). Editors. *Comprehensive Heterocyclic Chemistry II*, Vol. 4, pp. 1–126. Oxford: Elsevier Science.

[bb8] Key, J. A., Cairo, C. W. & Ferguson, M. J. (2008). *Acta Cryst.* E**64**, o1910. 10.1107/S1600536808028250 PMC2959342 21201119

[bb9] Macrae, C. F., Edgington, P. R., McCabe, P., Pidcock, E., Shields, G. P., Taylor, R., Towler, M. & van de Streek, J. (2006). *J. Appl. Cryst.* **39**, 453–457.

[bb10] Makam, S. R. & Yulin, L. (2004). *Tetrahedron Lett.* **45**, 6129–6132.

[bb11] Santos-Contreras, R. J., Ramos-Organillo, A., García-Báez, E. V., Padilla-Martínez, I. I. & Martínez-Martínez, F. J. (2009). *Acta Cryst.* C**65**, o8–o10. 10.1107/S0108270108040407 19129607

[bb12] Sheldrick, G. M. (2008). *Acta Cryst.* A**64**, 112–122. 10.1107/S0108767307043930 18156677

[bb13] Siemens (1996). *XSCANS* Siemens Analytical X-ray Instruments Inc., Madison, Wisconsin, USA.

[bb14] Vaqueiro, P. (2006). *Acta Cryst.* E**62**, o2632–o2633.

